# Liposomal Formulation of a PLA2-Sensitive Phospholipid–Allocolchicinoid Conjugate: Stability and Activity Studies In Vitro

**DOI:** 10.3390/ijms23031034

**Published:** 2022-01-18

**Authors:** Maria K. Kobanenko, Daria S. Tretiakova, Ekaterina S. Shchegravina, Nadezhda V. Antipova, Ivan A. Boldyrev, Alexey Yu. Fedorov, Elena L. Vodovozova, Natalia R. Onishchenko

**Affiliations:** 1 Shemyakin-Ovchinnikov Institute of Bioorganic Chemistry, Russian Academy of Sciences, ul. Miklukho-Maklaya 16/10, 117997 Moscow, Russia; mkobanenko4@gmail.com (M.K.K.); daria@lipids.ibch.ru (D.S.T.); nadine.antipova@gmail.com (N.V.A.); ivan@lipids.ibch.ru (I.A.B.); elvod@lipids.ibch.ru (E.L.V.); 2 Department of Chemistry, Lobachevsky State University of Nizhny Novgorod, Gagarin av. 23, 603950 Nizhny Novgorod, Russia; sc.katarina@yandex.ru (E.S.S.); afedorovnn@yandex.ru (A.Y.F.)

**Keywords:** colchicine, lipophilic prodrug, stimuli-responsive liposomes, protein corona

## Abstract

To assess the stability and efficiency of liposomes carrying a phospholipase A2-sensitive phospholipid-allocolchicinoid conjugate (aC-PC) in the bilayer, egg phosphatidylcholine and 1-palmitoyl-2-oleoylphosphatidylglycerol-based formulations were tested in plasma protein binding, tubulin polymerization inhibition, and cytotoxicity assays. Liposomes L-aC-PC10 containing 10 mol. % aC-PC in the bilayer bound less plasma proteins and were more stable in 50% plasma within 4 h incubation, according to calcein release and FRET-based assays. Liposomes with 25 mol. % of the prodrug (L-aC-PC25) were characterized by higher storage stability judged by their hydrodynamic radius evolution yet enhanced deposition of blood plasma opsonins on their surface according to SDS-PAGE and immunoblotting. Notably, inhibition of tubulin polymerization was found to require that the prodrug should be hydrolyzed to the parent allocolchicinoid. The L-aC-PC10 and L-aC-PC25 formulations demonstrated similar tubulin polymerization inhibition and cytotoxic activities. The L-aC-PC10 formulation should be beneficial for applications requiring liposome accumulation at tumor or inflammation sites.

## 1. Introduction

Colchicine is an antimitotic alkaloid isolated from the *Colchicum autumnale* plant. It binds the β subunit of tubulin, preventing the contacts between the α/β-tubulin heterodimers and microtubule elongation, which in turn results in the G2/M cell cycle arrest and ultimately apoptosis [1,2]. Anti-inflammatory effects of colchicine are mainly mediated by inhibition of neutrophil chemotaxis and cytokine release [3]. Low-dose colchicine has been approved to treat familial Mediterranean fever and acute gout flares. It is widely studied as an anti-inflammatory agent in various cardiovascular diseases [4,5,6]. Colchicine is considered as a promising anti-inflammatory drug to treat COVID-19 [7,8,9]: 36 relevant trials are registered at https://clinicaltrials.gov/as of 11 December 2021. Application of colchicine in cancer therapy is limited by its high general toxicity [10].

Heterocyclic allocolchicinoids exhibited lower acute toxicity yet comparable cytotoxicity in vitro and anticancer activity in vivo [11,12,13,14]. Incorporation of drugs in nanosized liposomal carriers allows to decrease systemic toxicity, first of all, due to the decreased volume of distribution [15]. Lipophilic derivatization can be adopted to utilize the bilayer compartment for the drug loading. The approach offers several benefits. (1) High loading capacity can be achieved matching that of DOXIL, and alike, where drug salt crystals are formed within the liposomes. (2) From the technology point of view, it requires no separation of the unencapsulated drug. (3) It offers the opportunity to utilize surface-active enzymes, e.g., phospholipase A2, to release the active drug. Since phospholipase A2 has been reported overexpressed in inflammation sites and in some tumors, this adds to the selectivity of such drug carrier.

**Figure 1:**
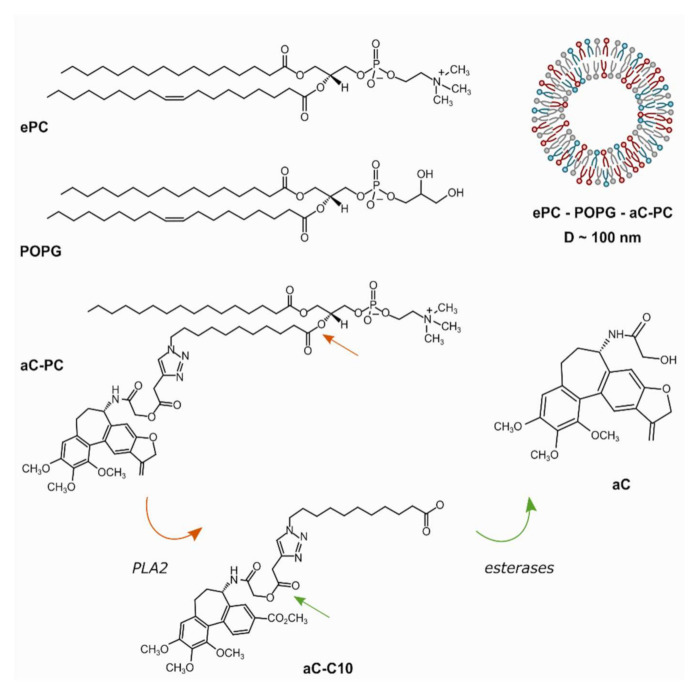
Schematic representation of a liposome with allocolchicinoid phospholipid prodrug (**aC-PC**) in the bilayer and matrix lipids used in the work, egg yolk-derived phosphatidylcholine (**ePC**, a representative structure is shown) and 1-palmitoyl-2-oleoylphosphatidylglycerol (**POPG**). The prodrug is subject to enzymatic hydrolysis by phospholipase A2 (PLA2) and non-specific esterases yielding the parent dihydrofuran allocolchicinoid (**aC**).

Earlier, a pair of allocolchicinoids, the carboxymethyl and dihydrofuran congeners, with enhanced cytotoxicity have been synthesized and used to generate phospholipase A2 (PLA2)-responsive phosphatidylcholine prodrugs [16]. The prodrugs were proven to be easily incorporated in egg phosphatidylcholine (ePC) liposomes. In the amount of 5 mol. % of the prodrug, no distortion of the bilayer structure was observed according to the surface pressure–area isotherms [16]. The dihydrofuran allocolchicinoid and its phospholipid prodrug (aC and aC-PC, respectively; Figure 1) have been slightly more cytotoxic (~5- and 2–5-fold, respectively) and produced more stable liposomes compared to the carboxymethyl counterpart as assayed on PANC-1, Colo-357, BxPC-3, and HaCaT cell lines [16]. Assuming there are hydrophobic regions covering up to 20% of the bilayer surface area [17] and taking into account optimized geometry of the allocolchicinoid [16], here we tested whether as much as 25 mol. % of aC-PC could be incorporated in the bilayer of the ePC liposomes.

In circulation, liposomes of pure ePC bind considerable amount of proteins gaining the so-called protein corona and are rapidly removed by mononuclear phagocyte system (also known as reticular endothelial system, RES) [18]. The presence of phosphatidylglycerol (PG) and cholesterol (Chol) has been shown to enhance liposome stability toward aggregation in the presence of plasma proteins [19]. Moreover, incorporation of 33% Chol in the bilayer (to create the liquid ordered lipid phase) and/or anionic lipids (e.g., GM_1_) is known to improve the blood-to-RES tissue ratio in mice by 1–2 orders of magnitude [20]. Thus, variation of 1-palmitoyl-2-oleoylphosphatidylglycerol (POPG), a commercially available liquid-phase anionic lipid, and cholesterol content in the ePC bilayer can be used to improve stability of aC-PC-loaded liposomes in circulation.

The aim of the work was to optimize the lipid composition of liposomes loaded with aC-PC in the bilayer for better shelf-life and circulation stability and higher loading capacity. The selected formulations were further explored with respect to the ability of the prodrug-loaded liposomes to inhibit tubulin polymerization and cell proliferation.

## 2. Results and Discussion

### 2.1. aC-PC Liposome Composition

In search for a stable liposomal formulation for aC-PC with high prodrug loading, ePC-based compositions supplemented with cholesterol and/or POPG were screened. Ordered liquid phase bilayers with over 30% cholesterol did not accommodate reliably more than 5 mol. % phospholipid prodrug, even in the presence of 20 mol. % POPG (data not shown). These compositions produced dispersions with average particle diameters exceeding 400 nm and high polydispersity indices (PDI), which is not acceptable for desired intravenous administration. Decrease of the cholesterol content down to 25 mol. % resulted in a stable formulation. Thus, we further explored formulations with 20 mol. % POPG and 10 mol. % aC-PC prodrug, either containing 25 mol. % cholesterol or not (Table 1). Additionally, we studied the possibility to include as much as 25 mol. % aC-PC prodrug in the ePC–POPG, 80:20, bilayer, which was estimated based on the area of hydrophobic regions reaching 20% of the bilayer surface area [17] and dimensions of the allocolchicinoid moiety [16].

The presence of 20 mol. % POPG in the bilayer of studied compositions ensured their strongly negative zeta potential (ZP) (Table 1). Addition of aC-PC resulted in a decrease of the absolute value of ZP, independently of the amount added or the matrix lipid composition (cholesterol-containing or not). Such partial screening of surface charge by aC-PC in the bilayer indirectly supports floating of the allocolchicinoid moiety on surface of the bilayer predicted basing on the lateral pressure profile [16]. Interestingly, judging by the evolution of liposome diameters upon storage (Table 1), colloidal stability of the 25% prodrug formulation was higher than that of the 10% prodrug liposomes. A possible reason could be that higher content of the prodrug results in optimal packing of the lipids in the presence of 20 mol. % POPG. Previously, “compensating” behavior has been observed in liposomes carrying equimolar amounts of a melphalan dioleoylglyceride prodrug and phosphatidylinositol (PI): such liposomes were the most stable with respect to interaction with serum albumin, compared to either prodrug or PI alone in the ePC-based bilayer, according to FT-IR [21].

### 2.2. aC-PC Liposome Stability in Plasma

**Figure 2:**
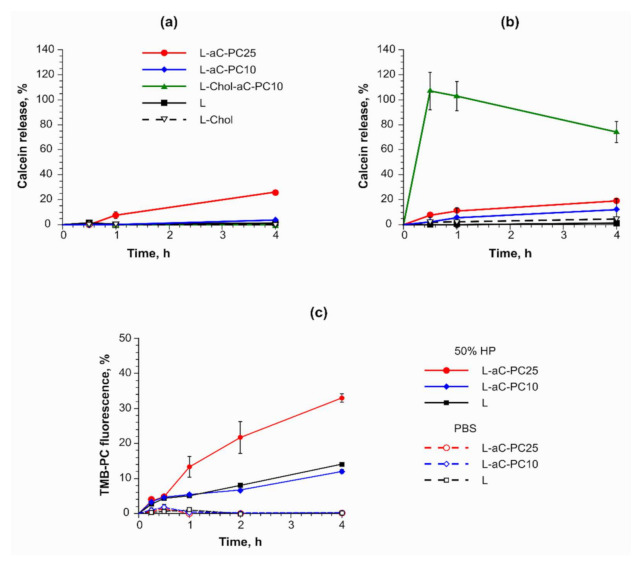
(**a,b**) Calcein release assay for cholesterol-free L-aC-PC10 and L-aC-PC25 liposomes and relevant control (L) and cholesterol-containing L-Chol-aC-PC10 and L-Chol control in (**a**) PBS and (**b**) 50% human plasma (HP). Mean ± SD are reported. *n* = 3. (**c**) FRET assay utilizing the TMB-PC and BCHB-PC pair of lipid bilayer probes in L-aC-PC10, L-aC-PC25, and control (L) liposomes in PBS and 50% HP. *n* = 2. Mean ± SE are reported. For sample designations, see Table 1.

As the first step, liposome stability in plasma was assessed in the calcein release assay [22]. The dye encapsulated in the liposomes at self-quenching concentration leaks in case bilayer integrity is disrupted, which is accompanied by fluorescence buildup. While slow leakage may be ascribed to both reversible pore formation in the bilayer and gradual rupture of liposomes, sharp rise of the fluorescence signal indicates destruction of the vesicular structure of liposomes. Liposome payload leakage in vitro has been demonstrated to correlate with in vivo release [23]. Three aC-PC formulations (see Table 1) maintained their integrity during incubation in PBS at 37 °C in the calcein release assay (Figure 2a). Both formulations containing 10 mol % aC-PC showed no significant difference from the prodrug-free controls. The least stable was the L-aC-PC25 sample, releasing 26.1 ± 0.5% of the entrapped dye over 4 h. In 50% human plasma (HP), cholesterol-free aC-PC liposomal formulations maintained their stability, while burst release of calcein from L-Chol-aC-PC10 was observed (Figure 2b, green line). Typically, the liquid ordered phase formed by cholesterol mixed with phospholipids binds less protein and the liposomes prepared thereof are more stable in circulation [24]. Our results suggest that cholesterol-containing fluid-phase bilayers poorly accommodate extraneous lipid–drug conjugates. Thus, in further experiments we compared liposomes containing 10 and 25 mol. % aC-PC in ePC–POPG-based bilayers.

To gain details of the bilayer behavior in the presence of plasma proteins, we used the Förster resonance energy transfer (FRET) assay with the tetramethyl-BODIPY-PC (TMB-PC, a donor) and bicyclohexyl-BODIPY-PC (BCHB-PC, an acceptor) probes incorporated in liposome membranes (Figure 2c). The probes have been designed to minimally disturb the bilayer [25]. While the donor and the acceptor, on average, stay close enough to each other in the bilayer, FRET is observed and no fluorescence of the donor is registered. With increasing distance between the donor and the acceptor, the efficiency of the energy transfer declines and the donor fluorescence grows. Nothing disrupted the interaction of the probes during liposome incubation in PBS for control, as well as both aC-PC, formulations (Figure 2c, dashed lines). The addition of plasma proteins caused gradual fluorescence build-up in all samples, evidencing increased distance between the probes. Thus, the bilayers could be destabilized by the processes of binding or embedding of proteins, releasing at least some of the bilayer-forming lipids into micelles or complexes with proteins. The process was the most pronounced in the case of the 25% aC-PC formulation.

### 2.3. Characteristics of the Protein Corona of aC-PC Liposomes

**Figure 3:**
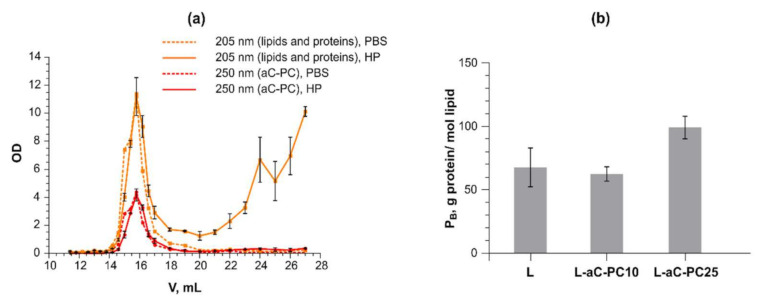
(**a**) Gel permeation chromatography elution profiles obtained for the L-aC-PC10 liposomes incubated with PBS (dashed lines) or 50% human plasma (HP; solid lines) through measurement of absorption at 205 (lipids and protein; orange) and 250 nm (aC-PC; red) in the fractions. (**b**) Protein binding values obtained for the joint liposome peak fractions upon concentrating via ultrafiltration. Mean ± SE are reported. *n* = 2. For sample designations, see Table 1.

To further compare the performance of the liposomal compositions in vitro, we analyzed protein binding (P_B_) values and relative amount of most common opsonins in the protein coronas formed on 10 and 25% aC-PC liposomes. To isolate the liposome–protein complexes, gel permeation chromatography (GPC) was utilized, as it is a mild technique allowing preserving the complexes and avoiding loss of the material as opposed to centrifugation. Figure 3a shows typical elution profiles of the L-aC-PC10 sample as monitored by absorbance at 205 (tracing lipids and peptide bonds of proteins) and 250 nm (tracing aC-PC) (Figure 3a). As separation is based on size, plasma particles (such as lipoproteins and exosomes) might have been present in the liposome-containing peak fractions [26]. To take this into account, a plasma–PBS control was assayed the same way as the liposomes.

Protein binding (P_B_, g protein/mol lipid) values obtained under in vitro settings have been shown to predict liposome blood residence times in vivo [24]. The lower the P_B_ value, the longer the liposomes stay in circulation and the more they accumulate at tumor and/or inflammation sites due to the enhanced retention and permeability (EPR) effect [27]. Despite the high storage stability of L-aC-PC25, P_B_ values were the highest for the formulation (99.1 ± 8.9 vs. 62.4 ± 5.6 g protein/mol lipid for the L-aC-PC10 liposomes; Figure 3b). Both values are high, predicting rapid removal of such liposomes from circulation, which is typical of anionic liposomes. The trend persisted upon subtracting of the protein eluting in the void volume fractions upon GPC of plasma control (incubated with PBS 1:1).

However, this manipulation compromised the significance of differences between the liposome compositions: (21 ± 16, 26 ± 12, and 65 ± 45 g protein/mol lipid for samples L, L-aC-PC10, and L-aC-PC25, respectively). Higher P_B_ value of L-aC-PC25 implies that the surface of such liposomes promotes protein binding compared to L-aC-PC10. The result is in line with somewhat lower stability of the L-aC-PC25 composition in plasma observed in calcein release and FRET assays (Figure 2). Higher protein binding by the L-aC-PC25 formulation together with higher fluorescence build-up in FRET assay can be interpreted as more probe (and, presumably, matrix lipids) leaving the bilayer to bind proteins.

**Figure 4:**
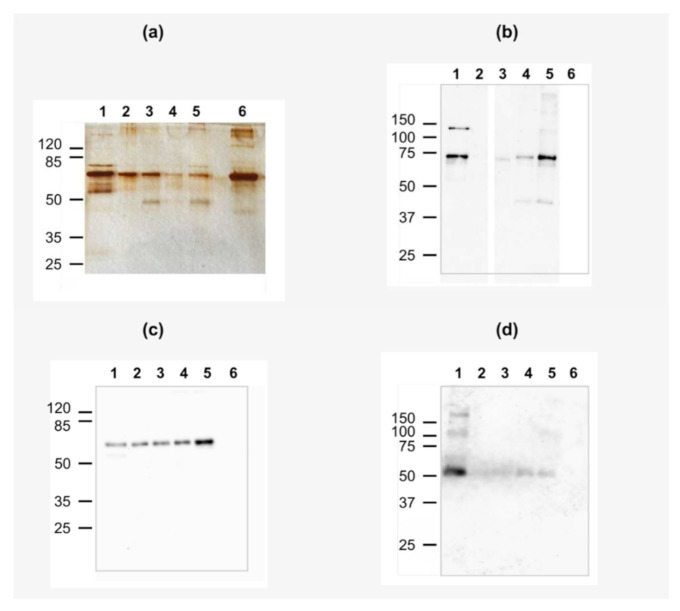
(**a**) SDS-PAGE of aC-PC and control liposomes. (**b**–**d**) Western blot (WB) of protein corona proteins with anti-C3 (**b**), anti-IgM (**c**), and anti-IgG (**d**) antibodies. Lanes 1, human plasma diluted 500-fold (positive control in WB); 2, plasma subjected to GPC control; 3, L; 4, L-aC-PC10; 5, L-aC-PC25; 6, HSA (negative control in WB). For liposome sample designations, see Table 1.

According to silver stained SDS-PAGE gels (Figure 4a), all liposomes carried a wide spectrum of proteins upon incubation in 50% human plasma. These are mostly mid-weight proteins, the most pronounced band probably corresponding to human serum albumin (HSA) in all coronas. aC-PC-free liposomes reproducibly carried the highest amount of proteins in the corona (Figure 4a, lane 3 vs. lanes 4 and 5). This contradicts the P_B_ values. The reason could be the uneven distribution of plasma proteins between chloroform–methanol, methanol–water, and interface during delipidization for different compositions of the corona. Notably, L-aC-PC25 carried the highest relative amount of ~75 and 45–50-kDa proteins. We used western blotting with antibodies against most prominent opsonins frequently identified in protein coronas of liposomes (complement factor C3 and immunoglobulins G and M) to identify the proteins.

As for factor C3, β chain of fragment C3b (~74 kDa) associated with all liposomal samples (Figure 4b, lanes 3–5). The highest amount of C3b and its cleavage products associated with the L-aC-PC25 liposomes. In the bilayer, the aC moiety of the aC-PC prodrug is considered to be forced out into the region of the lipid polar head groups [16]. Presumably, this exposes the nucleophilic groups in the aC-PC molecule, e.g., carbonyl of the linker, for the attack of the C3b thioester group [28]. Immunoglobulins M (IgM) were detected in all samples (Figure 4c, lanes 3–5). Indeed, natural (i.e., not immune) IgM against phospholipids are present in human plasma [29]. However, only the L-aC-PC25 liposomes bound IgM in the amount considerably exceeding the amount found in the control plasma sample subjected to GPC and delipidization similar to the liposomes (Figure 4c, lane 2). Little immunoglobulin G was detected in the protein coronas of the liposomes (Figure 4d). Thus, two major opsonins, factor C3 and IgM concentrate on surface of L-aC-PC25 liposomes. Deposition of opsonins on surface of liposomes promotes their internalization by blood phagocytes, monocytes, and to a lesser extent, neutrophils (e.g., [30]). Liposomes remaining in plasma are subject to the enhanced permeability and retention (EPR) effect, which promotes passive accumulation of 100–150-nm particles in tumor tissues and sites of inflammation [27]. Alternatively, liposomes with high plasma protein binding might be internalized by blood phagocytes, which retains the liposomes in circulation but makes them unavailable to tumor and/or inflamed tissues [31]. This interaction with circulating leukocytes may eventually lead liposomes to liver, lung, and spleen, yet might be beneficial if a population of blood cells is targeted. Particularly, the anti-inflammatory effects of colchicine are ascribed to its effect on neutrophils [3].

### 2.4. Tubulin Polymerization in the Presence of aC-PC Liposomes

**Figure 5:**
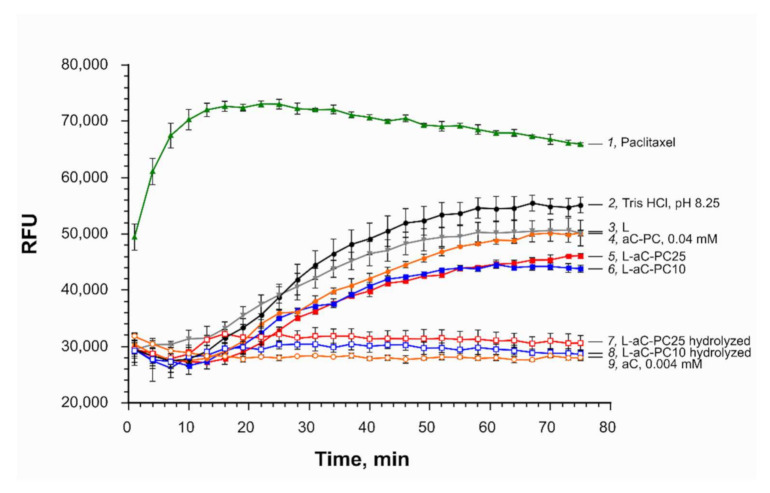
Tubulin polymerization assay. Effect of various liposomal samples on tubulin aggregation at 34 μM in tubulin polymerization buffer at 37 °C: *1*, paclitaxel, 3 μM, polymerization enhancer control (green triangles); *2*, Tris-HCl, pH 8.25, buffer (black circles); *3*, L, 0.4 mM total lipids (negative control; gray reversed triangles); *4*, aC-PC, 40 μM (orange filled circles); *5*, L-aC-PC25 (100 μM aC-PC; red filled squares); *6*, L-aC-PC10 (equivalent to 40 μM aC-PC; blue filled squares); *7*, L-aC-PC25 (100 μM aC-PC) hydrolyzed to aC (red empty squares); *8*, L-aC-PC10 (40 μM aC-PC) hydrolyzed to aC (blue empty squares); *9*, aC, 4 μM (orange empty circles). Mean ± SE are reported. *n* = 3. For sample designations, see Table 1.

To verify that the phospholipid prodrug retains the ability to inhibit tubulin polymerization, we tested the aC-PC in a cell-free tubulin polymerization assay. Free allocolchicinoid aC, 4 μM, inhibited tubulin polymerization completely (Figure 5, curve *9*). Either liposome formulation with a ~10-fold higher concentration of the active agent (40 or 100 μM aC-PC in formulations with 10 and 25% respectively, 0.4 mM total lipids), slowed down polymerization mildly (30–40% inhibition) (Figure 5, curves *5* and *6*). Lower inhibitory activity of liposomal formulations can be due to both lower binding affinity of the prodrug to tubulin compared to free aC and kinetics of liposome unloading and prodrug interaction with tubulin. Empty liposomes (0.4 mM total lipids; Figure 5, curve *3*) or the aC-PC phospholipid derivative (40 μM; Figure 5, curve *4*), applied in the form of micelles formed upon dissolution of the compound in DMSO and further dilution in buffer, caused an even less significant deceleration (by 16 and 24%, respectively). The effect of lipids on the rate of tubulin polymerization is probably due to the solubilization of the fluorescent reporter of the test system (DAPI, 4’,6-diamidino-2-phenylindole) and/or other components.

**Figure 6:**
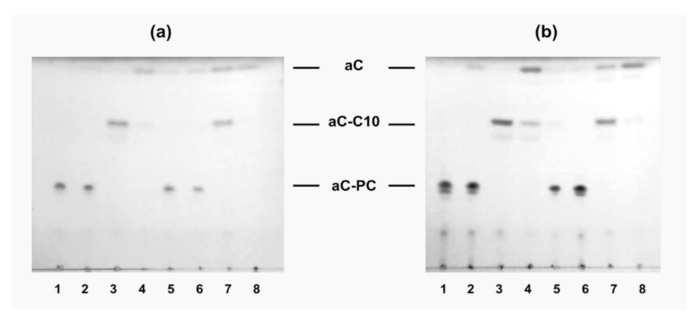
Thin layer chromatography of the L-aC-PC10 (**a**) and L-aC-PC25 (**b**) samples upon incubation in the absence of enzymes (tracks 1 and 5) and in the presence of porcine liver esterase (tracks 2 and 6), or phospholipase A2 (tracks 3 and 7), or both the esterase and phospholipase A2 (tracks 4 and 8) in Tris-HCl buffer, pH 8.25 (tracks 1–4) or 50% human plasma (tracks 5–8), at 37 °C for 4 h. aC, free allocolchicinoid; aC-C10, fatty acid derivative of aC; aC-PC, phospholipid prodrug. UV detection. For sample designations, see Table 1.

To test whether enzymatic release of free allocolchicinoid is necessary for the manifestation of microtubule destabilization activity by aC-PC, hydrolysis of the L-aC- PC10 and L-aC-PC25 samples by a combination of a phospholipase A2 (to hydrolyze the ester bond in the *sn*-2 position of the phospholipid derivative) and an esterase (to cleave the fatty acid moiety from the allocolchicinoid) was studied. The hydrolysis was followed by TLC with UV detection of aC-bearing derivatives (Figure 6). Incubation of the liposomes in the presence of porcine liver esterase (PLE) resulted in insignificant hydrolysis of the prodrug to free aC (Figure 6a,b, lanes 2), which was more pronounced in the case of the L-aC-PC25 formulation due to higher loading of the sample with the prodrug. PLA2, which is a surface-active enzyme, cleaved all the available prodrug in either formulation to lysophosphatidylcholine (not seen under UV) and fatty acid derivative of aC, aC-C10 (Figure 6a,b, lanes 3; see Figure 1 for formulae). Combination of the enzymes converted most of the prodrug to free allocolchicinoid, a fraction of it remaining in the form of aC-C10 (Figure 1) as is demonstrated by lanes 4 in Figure 6a,b. In 50% human plasma, minute amounts of aC were produced even in the absence of the enzymes, probably due to the inherent esterase activity thereof. Plasma also promoted the aC release from aC-C10, even in the absence of external esterase (compare lanes 3 and 7 in Figure 6a,b), as well as complete hydrolysis of the prodrug to free aC (lanes 4 vs. 8, Figure 6a,b). Presumably, plasma proteins promote disassembly of the liposomes, making the prodrug available for esterases.

Hydrolyzed samples inhibited tubulin polymerization completely in the cell-free assay, similar to free drug (Figure 5, curves *7* and *8*). Thus, to manifest their activity, aC-PC-containing liposomes are to be disassembled and the prodrug should be cleaved releasing free drug. Various (allo)colchicinoid prodrugs have been proposed thus far [2]. Many of the modification, while affecting the activity, require no release of free drug for the manifestation of microtubule destabilizing effects of the prodrugs. Lower microtubule-binding activity of lipophilic conjugates of allocolchicines, as well as complete loss of the activity for some of the derivatives, has been observed earlier [32]. However, in vitro, the prodrugs were rapidly processed by cellular enzymes, which even allowed surpassing the cytotoxic activity of some of the liposomal formulations of the prodrugs compared to free drug [31]. We further verified whether cytotoxic activity was compromised by phospholipid conjugation and loading of the prodrug into negatively charged liposomes.

### 2.5. Cytotoxicity Assessment

**Figure 7:**
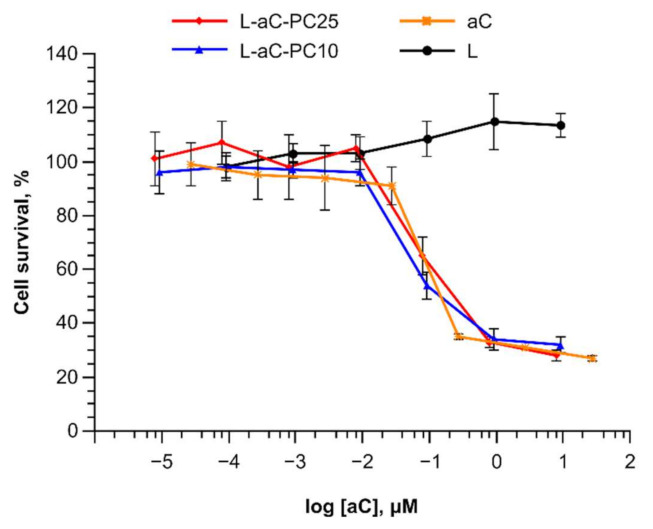
Cytotoxic activity of aC-PC liposomes against glioblastoma cells. L-aC-PC10, blue; L-aC-PC25, red; aC, orange; L, black. Mean ± SE are reported. *n* = 5.

Cytotoxicity of the aC-PC liposomes was assessed on the U87 human glioblastoma cells. The original tumor is aggressive and is characterized by high microtubule content, which makes microtubule-disrupting agents a promising treatment option [33]. Elevated levels of PLA2 group V are associated with poor prognosis for glioblastoma patients [34]. In the cytotoxicity tests, control prodrug-free liposomes stimulated cell growth at the highest concentrations, while either liposomal formulation exhibited cytotoxicity as high as that of the free drug (Figure 7). As reported by Sperry and co-workers [35], under low-glucose (2.5 vs. 25 mM as high glucose) conditions, palmitic acid supplemented at lower doses (50–100 μM) enhanced U87 cell proliferation, while inhibiting it at 200 μM. Thus, fatty acids derived from hydrolysis of liposomal phospholipids under conditions of the cytotoxicity assay (with the highest total lipid concentration used 100 μM) could promote cell proliferation. Destabilization of microtubules by free aC resulted in accumulation of cells in G2/M phase as estimated for several cell lines, which is the same manner of cell cycle arrest and apoptosis induction as colchicine [13]. We expect that liposomes carrying aC-PC prodrug release free aC, which then also follows the same mechanism of action. Meanwhile, liposome internalization and unloading require time. Typically, drug-loaded liposomes exhibit cytotoxicity lower than free drug by 1–2 orders of magnitude [15,36]. For colchicinoids, it is not the first report on liposomal formulations being as potent as the free drug [16,32]. Together with the data on tubulin polymerization inhibition, high cytotoxic activity of the aC-PC liposomes evidences availability of the prodrug to cellular enzymes and processing of the prodrug to yield the active compound, aC.

According to [37], glioblastoma multiforme (GBM) is characterized by an elevated level of cPLA2. If also true for U87, which is likely a glioblastoma [38], the following explanation of equal cytotoxicity of L-aC-PC10 and L-aC-PC25 can be suggested: L-aC-PC25 is less stable in the presence of serum proteins in the cultivation medium and releases some of its content before it enters the cell, however, inside cell the released prodrug gets hydrolyzed by cPLA2 (and not the surface-active sPLA2), thus the content of L-aC-PC25 is hydrolyzed faster than that of L-aC-PC10.

## 3. Conclusions

High drug loading capacity into liposomes may be achieved upon the drug conjugation with a phospholipid at the distal end of sn-2 fatty acid and loading the fluid-phase bilayer with the prodrug. Of note, the addition of cholesterol to the prodrug-containing compositions did not stabilize them against plasma protein-mediated disruption. Two liposomal formulations carrying 10 and 25 mol. % of an allocolchicinoid phospholipid conjugate in the ePC–POPG-based bilayer were proposed. Free allocolchicinoid release was achieved in an in vitro enzymatic assay and cytotoxicity experiments. No significant difference in the rate of prodrug release and liposome activity was observed in these two assays. Higher colloidal stability of the highly loaded formulation, L-aC-PC25, did not guarantee the stability in the presence of human plasma proteins: common plasma opsonins, C3 and IgM, were found to concentrate on the L-aC-PC25 liposomes. Tubulin polymerization assay evidenced the need for the prodrug to be hydrolyzed to free allocolchicinoid in order to manifest its microtubule destabilizing activity. Meanwhile, L-aC-PC10 with lower prodrug content, exhibited similar activity in tubulin polymerization inhibition and cytotoxicity assays, while being more stable in plasma than the L-aC-PC25 formulation. Thus, L-aC-PC10 should be beneficial for applications requiring liposome (passive) targeting to inflammation or tumor sites, while L-aC-PC25 could be optimized to target macrophages, specifically neutrophils, for systemic anti-inflammatory effect.

## 4. Material and Methods

### 4.1. Reagents and Equipment

Egg phosphatidylcholine (**ePC**) and cholesterol (**Chol**) were from Lipoid GmbH (Heidelberg, Germany); 1-palmitoyl-2-oleoyl-*sn*-glycero-3-phospho (1′-*rac*-glycerol) (**POPG**), from Avanti Polar Lipids, Inc. (Alabaster, AL, USA). (aR,5S)-*N*-(9,10,11-Trimethoxy-6,7-dihydro-5*H*-dibenzo[*a*,*c*]cyclohepten-5-yl-[2,3-*f*]-3′-methylene-2′-hydrofuranyl)2-hydroxyacetamide (**aC**) and its phosphatidylcholine derivative (**aC-PC**) were prepared as previously described [16]. 1,3,5,7-Tetramethyl-BODIPY-labeled phosphatidylcholine (TMB-PC) [39] and bis-cyclohexyl-BODIPY-labeled phosphatidylcholine (BCHB-PC) [40] were synthesized as previously described. Ethylene glycol bis (2-ethylamino) tetraacetate (EGTA), Tween 20, phenylmethylsulfonyl fluoride (PMSF), bee venom phospholipase A2, and porcine liver esterase were from Merck KGaA (Darmstadt, Germany); calcein (tetrasodium salt), from Serva; Tris; BSA (PanEko, Moscow, Russia); HSA; Sepharose CL-4B and Sephadex G-50 (Pharmacia, Framingham, MA, USA). Chloroform and other solvents were purified according to standard procedures.

Buffer compositions were as follows: phosphate buffered saline (PBS; KH_2_PO_4_, 0.2 g/L; NaH_2_PO_4_ × 2H_2_O, 0.15 g/L; Na_2_HPO_4_, 1.0 g/L; KCl, 0.2 g/L; NaCl, 8.0 g/L, pH 7.4); Tris-buffered saline (TBS; NaCl, 4.39 g; Tris, 3.03 g; H_2_O_dd_, 500 mL), pH 7.97; Tris-HCl, pH 7.0 (30 mM Tris); SDS-PAGE sample buffer (0.075 M Tris-HCl, pH 6.8, 10% glycerin, 2% SDS, 5% β-mercaptoethanol, 0.01% bromophenol blue).

Primary goat antibodies to human component C3 (ComplementTech, Tyler, TX, USA), polyclonal goat antibodies to immunoglobulins G and M (IMTEK, Moscow, Russia) were used. Secondary antibodies were rabbit antibodies to goat IgG conjugated with horse-radish peroxidase (Jackson ImmunoResearch, West Grove, PA, USA).

Blood samples from four healthy donor volunteers were collected in vacuum tubes over sodium citrate (Lab-Vac, Chengwu, China). Plasma was separated by centrifugation at room temperature for 10 min at 2000× *g* (Jouan BR4i, Thermo Fisher Scientific, Waltham, MA, USA). The supernatants were pooled, transferred into fresh tubes, and centrifuged at 600× g for another 10 min (CM-6M, ELMI, Riga, Latvia). Plasma aliquots were frozen in liquid nitrogen and stored at –70 °C. For the experiments, an aliquot of plasma was thawed at 37 °C and used immediately.

### 4.2. Liposomes

Liposomes (large unilamellar vesicles) were prepared by lipid film hydration followed by extrusion [16,36]. Mixtures of ePC, and POPG, and the aC-PC phospholipid prodrug in the required molar ratios were co-evaporated from solutions in chloroform in round-bottom tubes on a rotary evaporator. The lipid films were further dried on an Iney-4 (Institute for Biological Instrumentation, Russian Academy of Sciences, Pushchino, Russia) freeze dryer at 7 Pa and hydrated with PBS (unless otherwise indicated) at room temperature for 2 h with stirring. Then the mixtures were subjected to 5–7 cycles of freezing (N_2_ liquid)–thawing (+40 °C) and extruded through Whatman Nuclepore membrane filters (Cytiva, Marlborough, MA, USA) with calibrated pore size of 100 nm 20 times using an Avanti Polar Lipids (USA) mini-extruder. The resulting dispersions were stored at 4 °C and used for experiments within 3 days. To study the stability of the dispersions, they were stored at +4 °C for up to three weeks.

#### Dynamic Laser Light Scattering

To determine hydrodynamic diameter of the liposomes, the dispersions were diluted to a final lipid concentration of 50 μg/mL in PBS. The measurements were carried out on a Brookhaven Particle Analyzer 90+ (Brookhaven Instruments Corp., Holtsville, NY, USA; helium-neon laser, 633 nm, 90° angle), 3 cycles of 1 min, or Zetasizer Nano ZS (Malvern Panalytical, Ltd., Malvern, UK; 633 nm, 173° angle; provided by the BioImaging and Spectroscopy Core Facility of the Skolkovo Institute of Science and Technology, Moscow, Russia), 3 measurements of 10 cycles per sample. 

### 4.3. Stability of aC-PC Liposomes in the Presence of Human Blood Plasma

#### 4.3.1. Calcein Release

The stability of liposomes in the presence of human blood plasma was investigated using the dye leakage method as previously described [22]. To prepare liposomes with calcein in a self-quenching concentration, lipid films were hydrated with 80 mM calcein solution in PBS. After extrusion, unencapsulated calcein was separated from calcein-containing liposomes using size exclusion chromatography on a column with Sephadex G-50 (1.3 × 18 cm) equilibrated in PBS. An aliquot of liposome dispersions (200 μL) was applied to the column and after the void volume (~4.5 mL), fractions of 150–200 µL were collected. Fractions with the highest liposome content were pooled. Calcein concentration in combined fractions was determined by spectrophotometry (λ_max_ 504 nm, ε 74,000 M^–1^ cm^–1^).

An aliquot of calcein-containing liposomes (5–7 μL) was diluted with pre-heated (37 °C) PBS or 50% human blood plasma to a concentration of 10^–4^–10^–5^ M and incubated at 37 °C for 0, 1, or 4 h. The fluorescence intensity of calcein was determined using the temperature-controlled cell of an F-4000 (Hitachi, Japan) fluorescence spectrometer before and after the liposome lysis with 10 μL of 20% Triton X-100 solution added per 200 μL of dispersion, λ_ex_ 485 nm, λ_em_ 509 nm. The fraction of calcein released before the addition of the detergent was calculated using the formula:

(1) $$\mathrm{CR}=\;\left(\frac{I_{t}}{I{\;}_{max,\;t}}-\frac{I_{0}}{I_{max,\;0}}\right)\;\times \;\frac{I_{max,\;t}}{\left(I_{max,\;t}-I_{0}\right)}\times 100\%,$$

where $I_{max,\;0}$ is the fluorescence intensity upon Triton X-100 addition immediately after the liposome dilution; $I_{max,\;t}$, fluorescence intensity upon Triton X-100 addition to diluted liposomes after incubation for time *t*. The CR values obtained in triplicate were used to plot the relative increase in the fluorescence of calcein in function of the incubation time.

#### 4.3.2. Förster Resonance Energy Transfer (FRET) Assay

The stability of liposomes in the presence of human blood plasma was investigated using a pair of FRET probes as previously described [22]. For experiments with FRET, 0.5 mol. % TMB-PC, and 1.5% BCHB-PC were added to the bilayer at the stage of lipid film preparation. An aliquot of liposomes (25 μL) was incubated in PBS or 50% blood plasma (final lipid concentration 10^–4^ M) at 37 °C for 0, 1, or 4 h. The fluorescence intensity of TMB-PC was recorded in a temperature-controlled cell of a F -4000 (Hitachi) fluorescence spectrometer before and after liposome lysis as described above, λ_ex_ 470 nm, λ_em_ 505 nm. The TMB-PC fluorescence build-up was calculated using the formula:

(2) $$F_{B}=\frac{I_{t}-I_{0}}{I_{max}-I_{0}}\times 100\%,$$

where $I_{0}$ is the fluorescence intensity immediately after dilution of liposomes with PBS or 50% blood plasma; $I_{t}$ is the fluorescence intensity at time *t*; $I_{max}$, maximum fluorescence intensity after the addition of Triton X-100. Each point was measured in duplicate.

### 4.4. Characteristics of the Protein Corona of aC-PC Liposomes

#### 4.4.1. Determination of the Protein Binding (P_B_) Values

Liposomes (20 mM total lipids; 200 μL) were incubated with 200 μL of plasma at 37 °C for 30 min. After the incubation, proteolysis was stopped by adding 4 μL of 0.1 M PMSF solution in ethanol. The mixture was applied to a CL-4B Sepharose column (1.5 × 29 cm) and eluted with PBS. After the void volume of ~12 mL, fractions of 400 μL were collected. As a control, plasma was incubated with PBS, 1:1, was fractionated likewise. Liposome elution was monitored by absorbance at 205 and 250 nm using the NanoDrop One^C^ (Thermo Fisher Scientific, Waltham, MA, USA) instrument. To determine the amount of liposome-bound plasma protein, 6–7 fractions with the highest liposome content were pooled and concentrated to ~1 mL by ultrafiltration in VivaSpin 6 MWCO 50,000 concentrators for 45 min at 800× *g* (CM-6M, ELMI). In concentrated pooled fractions of the liposome–protein complexes, the amount of protein was determined using the modified Lowry procedure [41] and phospholipids using the enzymatic colorimetric phosphatidylcholine assay (Sentinel Diagnostics, Milan, Italy). Particularly, 3 μL of a fraction and 150 μL of the working enzyme solution (phospholipase D, >1500 U/L; choline oxidase, >7500 U/L; 4-aminoantipyrine, 1.2 mM; peroxidase, >7000 U/L; TES, 50 mM, pH 7.6; hydroxybenzoic acid 12 mM; EDTA, 1.3 mM; sodium azide, <0.1%) were added per well in a 96-well plate. The mixture was incubated at 37 °C for 10 min. Optical density was read at 540 nm using a Multiskan FC (Thermo Fisher Scientific, Waltham, MA, USA) microplate photometer. The amount of phosphatidylcholine in the samples was determined using the calibration curve for ePC dispersions in PBS. The P_B_ values were calculated as g protein/mol of lipids. The experiment was repeated twice with independent batches of liposomes.

#### 4.4.2. Identification of Common Opsonins in the Protein Corona

Concentrated pooled fractions of the liposome–protein complexes were delipidized as described in [42]. To 100 μL of the combined fractions, 400 μL of cooled methanol was added and the mixture was centrifuged for 3 min at 9000× *g* (Hamburg, Germany). To the solution, 200 μL of chloroform was added, vigorously stirred, and centrifuged for 3 min at 9000× *g*. To the mixture, 300 μL of water was added, vigorously stirred, and centrifuged for 4 min at 9000× *g*. Approximately 700 μL of the upper aqueous phase was discarded. Then, 300 μL of methanol was added to the residue and the mixture was centrifuged for 4 min at 9000× *g*. The supernatant was decanted, and the precipitate was evaporated to dryness on a rotary evaporator. The samples were dissolved in 36 μL of 2× reducing buffer (0.075 M Tris-HCl, pH 6.8, 10% glycerol, 2% SDS, 5% β-mercaptoethanol, 0.01% bromophenol blue), stirred, and boiled 2 × 2 min. SDS-PAGE was performed in 6% concentrating and 12% separating gels on a Mini Gel Tank (Thermo Fisher Scientific, Waltham, MA, USA) apparatus for 45 min at 200 V. The SigmaMarker (Sigma, St. Louis, MO, USA) and Precision Plus Protein^TM^ Dual Color Standards (Bio-Rad Laboratories, Inc., Hercules, CA, USA) kits were used as molecular weight markers. Proteins were visualized by silver staining or transferred to a PVDF membrane using the Mini Gel Tank (Thermo Fisher Scientific, Waltham, MA, USA) at 20 V for 60 min. After the end of the transfer, the membrane was washed with TBS and, to prevent non-specific sorption, incubated in 5% low-fat dry milk in TBS with 0.1% Tween 20 (TBS/T) for 1 h at room temperature. Then, the membrane was washed with TBS/T (3 × 5 min) and incubated with anti-IgG, IgM, or C3 primary antibodies in 0.5% BSA solution for 2 h at room temperature with stirring. The membrane was washed with TBS/T for 15 min and 3 × 5 min and incubated with horseradish peroxidase-conjugated secondary antibodies at 4 °C overnight. The membrane was then washed again with TBS/T 5 × 5 min. Immunodetection was performed using Clarity™ ECL Western Blotting Substrate (Bio-Rad) and VersaDoc 4000 (Bio-Rad).

### 4.5. Polymerization of Tubulin in the Presence of the aC-PC Liposomes

#### 4.5.1. Tubulin Assay

Liposomes carrying 10 or 25% of the aC-PC prodrug in the bilayer were prepared as described above by hydrating the film with 30 mM Tris-HCl, pH 7.0, 400 μM total lipids. The Fluorescence Based Tubulin Polymerization (BK011P) (Cytoskeleton, Inc., Denver, CO, USA) kit was used to assay the polymerization of tubulin in the presence of liposomes according to the manufacturer’s recommendations. To 5 μL of dispersions, 50 μL of tubulin 34 μM solution in polymerization buffer was added and the plate was placed in the Hidex Sense (Hidex, Turku, Finland) fluorescent plate reader. Fluorescence (λ_ex_ 330 nm, λ_em_ 460 nm) was recorded for 90 min at 37 °C with a 1-min interval between the measurements. Concentration of the prodrug in the assay well was 40 μM for L-aC-PC10 and 100 μM for L-aC-PC25. A 30 μM paclitaxel solution (5 μL; 3 μM in the assay well) was used as a microtubule assembly activator control, and 5 μL of buffer was used as a negative control. Stock solutions of aC-PC (40 mM) and aC (4 mM) were prepared in DMSO at 100× concentration. To prepare test solutions of aC-PC and aC, 5 μL of a solution in DMSO was added to 500 μL of Tris-HCl buffer, pH 7.0. DMSO concentration in the tubulin assay did not exceed 0.1%.

#### 4.5.2. Enzymatic Hydrolysis of the Phospholipid Prodrug

Liposomes with 10 or 25% aC-PC prodrug (50 μL) were incubated with 1 μM bee venom phospholipase A2 and 30 U/mL porcine liver esterase in Tris-HCl buffer, pH 8.25, in the presence of 1 mM CaCl_2_ at 37 °C. After 4 h, a 4-fold excess of EGTA was added to stop the reaction. Then, water was co-evaporated with 200 μL of *tert*-butanol. To the dry residue, 200 μL of the chloroform–methanol mixture, 1:1, was added. The phospholipid composition of the mixture was analyzed by TLC on aluminum sheets pre-coated with silica gel (Kieselgel 60_F254_, Merck); solvent system CHCl_3_–CH_3_OH–NH_4_OH_conc_, 65:25:4. The plates were visualized under UV light and developed using Vaskovsky’s molybdenum blue reagent [43]. Liposomes incubated with bee phospholipase A2 or porcine liver esterase separately, as well as in the absence of the enzymes, were used as controls. If necessary, an aliquot (15 μL) of the reaction mixture was frozen in liquid nitrogen and stored at −20 °C for subsequent analysis of the effect of prodrug hydrolysis products on tubulin polymerization. To test the effect of the components of the reaction mixture on tubulin polymerization, EGTA was added immediately after mixing the liposomes, enzymes, and CaCl_2_. In parallel, similar incubations were carried out in the presence of 50% human blood plasma.

### 4.6. Cytotoxic Activity of the aC-PC Liposomes

The U87 human glioblastoma cells were grown in the DMEM/F-12 medium (~17 mM glucose) supplemented with 10% FBS, 2 mM glutamine, 100 U/mL penicillin, and 100 μg/mL streptomycin (PanEko), and incubated overnight at 37 °C in the atmosphere of 5% CO_2_. They were dissociated with Trypsin-Versene solution, transplanted into the wells of a 96-well plate, 8 × 10^3^ cells per well, and incubated for 24 h. Then, serial 10× dilutions of the aC-PC liposomes from 10^–4^ to 10^–11^ M (aC-PC concentration) in PBS were added 10 μL per 100 μL medium. A standard solution of aC in DMSO was diluted to a concentration of 10^–4^ M in PBS and used as control. The cytotoxic effect was assessed 5 days later using the alamarBlue^TM^ Cell Viability Reagent (Thermo Fisher, Waltham, MA, USA).

## Figures and Tables

**Table 1:** Studied liposome compositions and their characteristics.

Composition	Sample ID	ZP, mV *	Day 1		Day 7		Day 21	
			D_H_, nm	PDI	D_H_, nm	PDI	D_H_, nm	PDI
ePC–POPG 80:20 *	L	–59.2 ± 1.4	111.1 ± 34.1	0.094	112.9 ± 27.1	0.057	107.3 ± 29.2	0.074
ePC–Chol–POPG 55:25:20 *	L-Chol	–60.1 ± 0.6	ND	ND	ND	ND	ND	ND
ePC–POPG–aC-PC 70:20:10 *	L-aC-PC10	–49.5 ± 0.8	121.1 ± 34.0	0.079	159.3 ± 70.6	0.196	147.7 ± 52.7	0.127
ePC–POPG–aC-PC 55:20:25 *	L-aC-PC25	–50.2 ± 0.8	109.9 ± 22.6	0.042	111.5 ± 31.11	0.078	126.9 ± 28.4	0.050
ePC–Chol–POPG–aC-PC 45:25:20:10 **	L-Chol-aC-PC10	–54.9 ± 0.5	97.4 ± 21.3	0.055	105.7 ± 28.1	0.071	ND	ND

* Data obtained using Nanosizer ZS (Malvern Panalytical, Ltd., Malvern, UK), Z-average ± PDI width and ZP average ± SE are reported. ** Data obtained using 90Plus (Brookhaven Instruments) analyzer, average diameter ± half-height half-width are reported. ND, not determined.
